# Study of biological safety of camel milk after treatment with different antibiotics

**DOI:** 10.1371/journal.pone.0321807

**Published:** 2025-04-22

**Authors:** Zauresh Bilal, Farida Amutova, Zaira Kabdullina, Dariga Utemuratova, Askar Kondybayev, Shynar Akhmetsadykova, Zhaidar Musayev, Nurlan Akhmetsadykov, Bernard Faye, Gaukhar Konuspayeva

**Affiliations:** 1 Biotechnology Department, Al-Farabi Kazakh National University, Almaty, Kazakhstan; 2 LLP “Scientific and Production Enterprise Antigen”, Almaty, Kazakhstan; 3 Horse and Camel Breeding Department, LLP “Kazakh Research Institute for Livestock and Fodder Production”, Almaty, Kazakhstan; 4 Center of International Cooperation on Agriculture Research for Development–CIRAD, UMR SELMET, Campus International de Baillarguet, Montpellier, France; Kerman University of Medical Sciences, IRAN, ISLAMIC REPUBLIC OF

## Abstract

The use of antibiotics in camels is generally based on the doses applied to cattle, despite the known differences in plasma pharmacokinetics between camel and cattle. The demand for camel milk increased due to the belief that traditional camel farming practices produce safe milk with health benefits. For assessing the importance of antibiotic residues in camel milk and to propose a convenient withdrawal period, a trial was conducted on 10 lactating camels (7–12 years old; 450 kg life weight (LW), 7–8 liter of milk production per day) at mid-lactation receiving an injection of 40 mL of Pen-strep® (benzylpenicillin-procaine 200,000 IU/1 ml and dihydrostreptomycin sulfate 200 mg/1 ml) and Nitox® (oxytetracycline dihydrate 200 mg/1 ml). The antibiotic residues were measured in the milk 30 min after injection then on day 1, 2, 3, 7, 9, 14, 19, 24 and 56 by using HPLC-MS/MS. Results showed that penicillin’s mean residual concentration (0.3 ± 0.013 mg/kg) remained 7.5 times above the maximum residue limit (MRL) even on 56th day. In contrast, streptomycin fell below the MRL within 30 minutes and averaged 1 µg/kg by day 56. Oxytetracycline levels exceeded the MRL (0.1 mg/kg) until day 14 but dropped to a safe level of 0.018 ± 0.01 mg/kg by day 24. In conclusion, the decline in streptomycin concentration post-injection appeared rapid and efficient, the elimination of penicillin and tetracycline was slow. These preliminary results lead to considering the necessity to adapt the waiting time to the dairy camel.

## Introduction

Camel breeding plays a crucial role in supplying milk and meat products in many arid and semi-arid parts of the world. Camels, due to their interactions with other livestock species, wild animals, and the practice of seasonal transhumance, are susceptible to a variety of diseases. Moreover, the intensification of camel husbandry, coupled with suboptimal management practices, has raised significant food safety concerns. In such extensive or intensive context, diseases like mastitis, genital or respiratory infections, abscess and bacterial skin diseases could increase. These practices include the uncontrolled use and unauthorized sale of veterinary drugs, self-medication, use of wrong veterinary drugs and overdosing [1–3]. Such actions can lead to drug residues entering the food chain and contribute to the development of antibiotic resistance in harmful microorganisms that pose risks for both animals and humans [4,5].

Consequently, there is a global need for studies on the pharmacokinetics and metabolism of veterinary drugs in camels, as these processes are still relatively under-investigated in comparison to other livestock animals [2,6]. Observing proper withdrawal period is crucial for minimizing harmful residues [7], but this period could change according to the characteristics of the antibiotic used, the pharmacokinetics of the product and the physiology of the treated animal [8].

Current practices often apply withdrawal periods similar to those for cows, despite camels’ slower pharmacokinetics and metabolism [9]. The limited pharmacokinetic studies in camels [10–12] underscores the necessity for targeted research. Moreover, most of the studies in camel were focused on blood residues and not on milk. Existing studies have focused on monitoring residues of oxytetracycline and β-lactam in raw camel milk [13], but comprehensive investigations regarding biosafety remain scarce. Moreover, a variety of analytical methods are used, convenient, leading to questionable conclusions.

To address these challenges, the objective of our trial in Kazakhstan was to determine the necessary withdrawal period following antibiotic injection in camels. We focused on quantifying residues belonging to three different antibiotics (oxytetracycline, penicillin and streptomycin) in camel milk over an extended period post-treatment. Thus, the present preliminary study would contribute to providing specific recommendations for implementing an organic label for camel products, ensuring consumer safety and addressing the spread of antibiotic-resistant bacteria [14].

## Materials and methods

### Animals’ treatment

Camel antibiotics treatment was conducted strictly in accordance with ethics committee guidelines of Co Antigen LTD (protocol 10, from October 18, 2022) and based on the order of the Minister of Health of the Republic of Kazakhstan dated November 19, 2009, No. 744. The studied animals were housed on the same farm – Makhanov farm, Akshi village, Almaty region, Kazakhstan to avoid differences in diet and management. The protocol was explained to the owner who gave his verbal consent. Ten dromedary camels (*Camelus dromedarius*) of Aruana breed camels were used in this experiment and shared into two groups of five camels randomly. The first group received an intramuscular single injection in the hindleg of “Nitox ®200” (ImmCont GmnH, Germany) with active compound: oxytetracycline dihydrate 200 mg mL^-1^ (0.1 mL kg^-1^ BW). The second group of 5 camels received the injection in the same anatomic place of “Pen-Strep 400LA” (Nita-Pharm, Russia) with active compounds: procaine penicillin G 200,000 IU mL^-1^ and dihydrostreptomycin sulfate 200 mg mL^-1^. The injections were achieved by veterinary practitioner. All camels were aged from 7 to 12 years, with a relatively equal lactation period, weighing 450–500 kg, and a similar milk production of 7–8 L/day. The camels were manually milked twice daily, with all milk discarded and not utilized for human consumption. Milk samples for analysis were collected during the evening milking session, typically conducted between 5:00 and 6:00 p.m. Immediately, at the end of milking, a part of the milk recipient was poured in a sterile bottle of 0,5 L. Milk sampling was carried out in the autumn-winter period (from November 2022 to January 2023). Samples were collected from the middle phase of the milking process. The camels were all on natural pasture (dominated by grass and shrubs as *Artemisia sp*., *Alhaji maurorum*, *Caragana korshinskii* and *Salsola tragus*), all over the year, grazing in a circle of approximatively 5 km around the farm, and they did not receive supplementary food. They spent the night in an enclosure with their calves and are in pasture from 9 a.m. after morning milking to 3 p.m. for the second milking, the calves remaining in the enclosure. During the experiment (autumn-winter), the temperature was -5°C -16°C. All the camels were deworming in summer time (August), and were in good health all over the experiment.

After injection by antibiotic drugs, milk samples were collected after the morning milking in following time intervals: 30 min, 24 hrs., 48 hrs., 7 days, 9 days, 14 days, 19 days, 24 days and 56 days. Collected milk samples were then analyzed for antibiotic content by HPLC-MS/MS as described below.

### Camel milk sample preparation

After sampling, camel milk was placed in a freezer and stored at -20⁰ C until HPLC analysis. Before extraction according to Santosh et al, 2017 [15], milk samples were placed at room temperature until fully thawed and gently stirred at least 3 times.

### Preparation of a buffer solution for oxytetracycline extraction

For preparation, 96.5 mL of sodium phosphate solution (0.2 mol L^-1^) and 106.5 mL of citric acid solution (0.1 mol L^-1^) were poured into 200 mL volumetric flask. To establish a pH of 4.6, 0.6 mL of EDTA solution (0.5 mol L^-1^) was added and mixed thoroughly.

### Preparation of internal standard solution based on demeclocycline

For accurate quantification of tetracycline group of antibiotics, demeclocycline was used as internal standard (IS) proposed in standard methodology of GOST 33526-2015 [16]. The demeclocycline (IS) solution was prepared by measuring 10 mg of demeclocycline hydrochloride (purity ≥ 98%, Sigma Aldrich, catalog №D6140, powder, 1 g) and dissolved in 5 mL of high-purity methanol. After ultrasonication for 1 min., the volume was adjusted to 10 mL with methanol, resulting in a concentration of 10 mg mL^-1^. Subsequent dilutions with methanol yielded a solution with final concentration of 1 μg mL^-1^. A solution with this concentration was used as an internal standard (IS) in analysis of oxytetracycline in camel milk.

### Camel milk extraction for penicillin

Extraction of penicillin in milk was performed according to GOST R 54904-2012 [17]. Briefly, one mL of milk sample was transferred to 15 mL falcon tube. Then, 4 mL of acetonitrile were added, and the sample was vortexed during 15 min and further centrifuged at 4000 rpm for 15 min at 4⁰C. Upper layer was transferred to another falcon tube and evaporated under nitrogen stream at 40⁰C up to 100µL. Then, 2 mL of deionized water was added to the tube with sample and vortexed during 2 min. Obtained extract was then extracted using SPE as described below.

### Carrying out solid phase extraction of penicillin

The SPE cartridge (CNWBOND LC-C18 SPE Cartridge, 2 g, 10mL) was preconditioned with 2 mL of 50% acetonitrile and equilibrated with 2 mL of deionized water. Then a sample of camel milk, previously prepared as described above, was transferred to SPE cartridge. Penicillin was eluted from the sorbent using 2 mL of acetonitrile. The resulting eluate was evaporated on a heating module in a stream of nitrogen under vacuum at a temperature of 40⁰C, then redissolved in 1 mL of water, filtered through 0.2 μ syringe filter, and analyzed on HPLC.

### Camel milk extraction for streptomycin

Extraction of streptomycin in milk was performed according to Santosh Kapil et al., 2017 [15]. Milk sample (1 mL) was taken and 10% Trichloroacetic acid (0.4 mL) added and vortexed for 5 minutes, followed by centrifugation at 4000 rpm for 5 minutes. Supernatant collected and filtered through 0.2 μ syringe filter and injected for LC-MS/MS analysis.

### Camel milk extraction for oxytetracycline

Protocol of the methodology for extraction of oxytetracycline in milk was adapted from GOST 33526-2015 [16]. Briefly, one gram of milk sample was placed in 50 mL centrifuge tube, and 100 µL of internal standard (1 μg mL^-1^) was added and mixed. Then, 3 mL of acetonitrile (HPLC grade) were added and vortexed for 3 min. After settling the samples for 15 min., centrifugation was performed at 4⁰ C at 4000 rpm for 20 min. The resulting aqueous fraction was transferred to another centrifuge tube and evaporated to 1 mL on a heating module in nitrogen stream (Hanon HN200 Sample Concentrator) at temperature of 40⁰C. Five ml of a buffer solution (as mentioned above) was added to the resulting residue and stirred, then another 5 ml of a buffer solution was added and placed in an ultrasonic bath for 2 min.

### Carrying out solid phase extraction of oxytetracycline

The SPE cartridge (CNWBOND LC-C18 SPE Cartridge, 2 g, 10 mL) was preconditioned with 6 mL of 50% methanol and equilibrated with 6 mL of deionized water. Then a sample of camel milk, previously prepared as described above, was transferred to SPE cartridge, and washed again with 6 mL of deionized water. Oxytetracycline was eluted from the sorbent using 6 mL of methanol. The resulting eluate was evaporated on a heating module in a stream of nitrogen under vacuum at temperature of 40⁰C, then redissolved in 1 mL of methanol and used for further analysis on HPLC.

### HPLC-MS/MS conditions

Analysis of milk samples were performed on HPLC system (Agilent 1260 Infinity LC, Germany) equipped with ion trap mass-spectrometer (IT-MS) amaZon SL (Bruker Daltonik GmbH, Germany). Separation of antibiotics was performed on HPLC packed column Discovery HS C18 with parameters 150 mm x 2.1 mm, 3µm (Supelco). Different HPLC programs were implemented for different antibiotic families:

1. For penicillin: Phase A was a deionized water and Phase B was HPLC grade methanol. HPLC was programmed as follows: from 0 to 0.5 min phase B was 25% and then increased to 100% for 4 min. Phase B was 100% up to 6.5 min and then it decreased to 25%. Injection volume – 50 µL. Column temperature – ambient.
2. For streptomycin: Phase A was a 0.5% formic acid in water and Phase B was a 0.5% formic acid in acetonitrile. Gradient mode was set as follows: from 0.01 to 0.5 min phase B was 5% and then increased up to 30% for 1min. It was 30% from 1 to 2 min. Then, phase B was decreased up to 5% for 3.5min. Injection volume – 50 µL. Column temperature – ambient.
3. For oxytetracycline: Phase A was a 0.5% formic acid in water and Phase B was 0.5% formic acid in acetonitrile. Separation was performed in gradient mode with 0% to 55% of phase B for 25 min. Flow rate was 0.5 mL/min. Injection volume – 50 µL. Column temperature – ambient.

Mass spectrometer was set in enhanced resolution and MS/MS mode. Scanning of ions was performed in positive mode (for oxytetracycline and streptomycin) and negative mode (for penicillin). The scan range of mass was set from 150 to 600 m z-1. Identification and quantitative analysis of antibiotics was done in Compass 1.3 software package for controlling the mass spectrometer and HPLC system. For this, parent and fragmented ions were used: Oxytetracycline (461.1, 426.1 and 444.2), penicillin (335, 217 and 202) and streptomycin (582.3, 263.2 and 390.2).

Oxytetracycline, penicillin and streptomycin values of limit of detection (LOD) were 0.009, 0.014 and 0.023 mg kg^-1^ respectively; the limits of quantification for the same antibiotics (LOQ) were 0.027, 0.042 and 0.069 mg kg^-1^, and recoveries % of analysis were 96.30%, 84.70% and 85.10% respectively (Table 1).

**Table 1:** Analytical performances of the method.

Antibiotic	Concentration range, mkg/L	Linear equation	R^2^	LOD, mkg/L	LOQ, mkg/L	Recoveries, %
Oxytetracycline	25-500	y=0.0188*x	0.9998	9.16	27,8	96.30%
Peniciline	50-500	y=2682.9*x	0.9941	13.8	41.8	84.70%
Streptomycine	50-500	y=98383*x	0.9953	22.7	68.9	85.10%

LOD – limit of detection.

LOQ – limit of quantification.

### Statistical analysis

The objective being to assess the kinetic of residue, the statistical analyses are limited to descriptive methods, i.e., the calculation of mean, standard-deviation and median regarding the data of all camels at each sampling time. Due to the lack of normality of the data, the non-parametric test of Kruskall-Wallis was applied to determine the time differences. The software used was XLstat (Addinsoft, 2024 ©).

## Results

**Fig 1:**
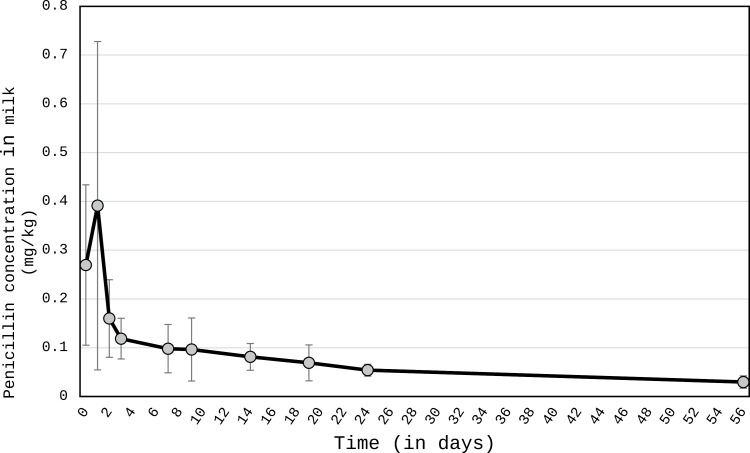
Kinetic of the residual penicillin concentration in camel milk after injection (in mg kg^-1^). The error bars represent a standard deviation.

**Fig 2:**
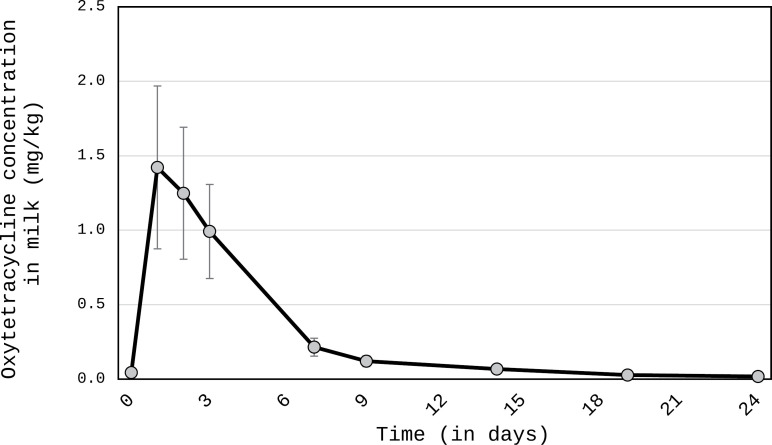
Kinetic of the residual oxytetracycline concentration in camel milk after injection (in mg kg^-1^). The error bars represent a standard deviation.

The kinetic was highly different according to the type of molecule. Generally, all kinetic data on residual antibiotic concentrations in camel milk after injection are demonstrated in Figs 1 and 2 as well as in Tables 2–4. In addition, in supplementary data the chromatograms for the samples and the standards are reported.

**Table 2:** Residual penicillin concentration in camel milk after injection (in mg kg^-1^).

Days after injection	0,02	1	2	3	7	9	14	19	24	56
**C1**	0.158	0.155	0.097	0.125	0.111	0.072	0.086	0.046	0.050	0.041
**C2**	0.237	0.140	0.097	0.074	0.078	0.061	0.075	0.085	0.070	0.023
**C3**	0.222	0.402	0.126	0.078	0.040	0.062	0.069	0.068	0.058	0.038
**C4**	0.173	0.297	0.283	0.149	0.087	0.074	0.051	0.025	0.037	0.010
**C5**	0.558	0.962	0.196	0.167	0.174	0.212	0.125	0.121	0.054	0.036
**Mean**	0.270	0.391	0.160	0.119	0.098	0.096	0.081	0.069	0.054	0.030
**SD**	0.164	0.337	0.080	0.042	0.050	0.065	0.027	0.037	0.012	0.013
**Median**	0.222	.0.297	0.126	0.125	0.087	0.072	0.075	0.068	0.054	0.036

C1 to C5 mean camel ID.

**Table 3:** Residual streptomycin concentration in camel milk after injection (in mg kg^-1^).

Days after injection	0,02	1	2	3	7	9	14	19	24	56
**C1**	<LOQ	<LOQ	<LOQ	<LOQ	<LOQ	<LOQ	<LOQ	<LOQ	<LOQ	<LOQ
**C2**	<LOQ	<LOQ	<LOQ	<LOQ	<LOQ	<LOQ	<LOQ	<LOQ	<LOQ	<LOQ
**C3**	0.139	0.070	<LOQ	<LOQ	<LOQ	<LOQ	<LOQ	<LOQ	<LOQ	<LOQ
**C4**	<LOQ	<LOQ	<LOQ	<LOQ	<LOQ	<LOQ	<LOQ	<LOQ	<LOQ	<LOQ
**C5**	0.264	0.155	0.083	<LOQ	<LOQ	<LOQ	<LOQ	<LOQ	<LOQ	<LOQ
**Mean**	0.095	<LOQ	<LOQ	<LOQ	<LOQ	<LOQ	<LOQ	<LOQ	<LOQ	<LOQ
**SD**	0.107	<LOQ	<LOQ	<LOQ	<LOQ	<LOQ	<LOQ	<LOQ	<LOQ	<LOQ
**Median**	<LOQ	<LOQ	<LOQ	<LOQ	<LOQ	<LOQ	<LOQ	<LOQ	<LOQ	<LOQ

C1 to C5 mean camel ID. Limit of quantification (LOQ) was 0.07 mg kg^-1^.

**Table 4:** Residual oxytetracycline concentration in camel milk after injection (in mg kg^-1^).

Days after injection	0.02	1	2	3	7	9	14	19	24
**C1**	0.026	1.422	1.147	0.986	0.170	0.085	0.070	0.019	0.012
**C2**	0.058	0.562	0.611	0.810	0.156	0.086	0.058	0.045	0.021
**C3**	0.051	1.759	1.491	1.279	0.277	0.169	0.102	0.027	0.017
**C4**	0.071	1.361	1.188	0.569	0.187	0.122	0.056	0.024	0.033
**C5**	0.013	2.005	1.803	1.315	0.281	0.139	0.050	0.024	0.006
**Mean**	0.044	1.422	1.248	0.991	0.214	0.120	0.067	0.028	0.018
**SD**	0.02	0.55	0.44	0.32	0.06	0.036	0.02	0.01	0.01
**Median**	0.051	1.422	1.188	0.986	0.187	0.122	0.058	0.024	0.017

C1 to C5 mean camel ID.

It is recalled that, according to EU, Eurasian Customer Union and Chinese legislation on human food safety MRL for penicillin, streptomycin and oxytetracycline are 0.004 mg kg^-1^, 0.2 mg kg^-1^ and 0.1 mg kg^-1^ respectively [18,19].

For penicillin, the maximum residue in camel milk occurred 24 h after injection, but with high variability between animals (from 0.16 to 0.96 mg kg^-1^). Inter-animal variability decreased significantly from 48 h. For one camel among five, however, the maximum concentration was observed 30 min after treatment while for another camel, the same concentration was observed 30 min and 24h after (Fig 1, Table 2). Finally, the concentration significantly dropped after 24 h. However, the average concentration of penicillin residue (0.300 ±0,013 mg kg^-1^) remained above the threshold, reaching 7.5 times the Maximum Residue Limit (MRL), or 0.004 mg kg^-1^, even at 56 days of the experiment (Fig 1).

The values of residues were significantly different (P<0.05) between in one side the first five sampling times (from 30 min to 7 days) and in another side, the five last sampling times (from 9 to 56 days).

Contrary to penicillin, the residual concentration of streptomycin declined rapidly after 30 min after injection was below LOQ (0.07) and consequently below MRL (0.2 mg kg^-1^) (Table 3). Only one camel among five had to wait 24 h to present values below 0.2 mg kg^-1^.

The main time difference occurred between the six first time sampling (30 min to 9 days) and the four last sampling (P<0.05).

The pattern of oxytetracycline was comparable to that of penicillin with a maximum residue after 24 h (1.42 ±0.55 mg kg^-1^) and overpassing MRL (0.1 mg kg^-1^) up to the 14th day on average after injection (Fig 2, Table 3). Two camels among five reached the MRL on the 9th day with one had to wait the 19th day. However, on 24th days, all samples contained residues between 6 and 33 µg kg^-1^ with mean concentration 0.018±0,01 mg kg^-1^ (Fig 2) demonstrating a safe level.

The values of residues were significantly different (p<0.001) in the sampling time from 1 to 3 days compared to the values reported at 30 min, then from 6 to 9 days.

## Discussion

### Methodology of detection in milk

Over the last decades, several groups of analytical methods have been used for quantifying antibiotics residues in milk. These include chromatographic, immunological, microbiological methods and express tests. Chromatographic methods are the most frequent as it was common for 17 works investigating antibiotics residues in milk (48.6%) [20–36]. Indeed, a variety of chromatographic methods such as high-pressure liquid chromatography (HPLC), thin-layer chromatography (TLC) could be applied for qualitative determination of different families of antibiotics in milk and other food products. Immunological methods including ELISA, Beta Star Combo were applied in 10 studies [22,23,30,31,37–42] followed by microbiological methods founded in 6 studies [24,34,43–46]. Other techniques based on express tests such as Delvotest and Acidification test could be used also for detection of antibiotics and were mentioned in 6 papers [28,41,47–50]. The use of HPLC for determining the antibiotic residues was regarded as the most convenient analytical method and was commonly accepted nowadays by the scientific community [51,52].

### Comparative pharmacokinetics of antibiotics

In our study five healthy camels only were used per group. In most part of the pharmacokinetics studies, from 3 to 6 camels are taken due to the availability and specificity of camel breeding [11,53–55].

There are few comparative studies regarding the pharmacokinetic of antibiotics in camel milk, contrary to serum or plasma investigations [56]. In a rare reference, Laraje et al., (2006) investigated the marbofloxacin residue in cow and camel blood by high-performance liquid chromatography method, concluding that the pharmacokinetic in camel was characterized by a higher maximum concentration, a more rapid absorption after injection and a longer terminal half-life than in cow, confirming the specificity of camel metabolism [12]. Similar observations were done long time ago by Oukessou et al., (1990): comparing sheep and camel [55], the mean residence time (MRT) of benzylpenicillin in plasma was double in dromedary (27.34 ±1.38 min) compared to sheep (14.95 ± 4.16 min) leading to conclude for a slower elimination of the drug in dromedary camel. In Pakistan, Al-Nazawi, (2003) comparing kinetics of long-action oxytetracycline in camel, sheep and goat, came to the same conclusion, with a slower plasma kinetic in camel compared to the other species [57]. Slow elimination of cefotaxime was also observed in a pharmacokinetic study involving three dromedary camel calves of 3 months by single intravenous injection [54].

The reasons explaining such specificity are probably multifactorial as stated by Ali et al., (1996) [58]. Drug metabolism is linked to kidney and liver functions that have peculiar characteristics in relationships to the ability of camel to survive in harsh conditions, especially in case of dehydration [10]. Moreover, the enzymatic system in camel, contributing to the metabolization of drugs may be deficient or lacking as it has been tested in a comparative study regarding paracetamol metabolization [59]. Other factors as the injection route (IV, IM), the individual status of the animal (gender, age) or health conditions could influence the pharmacodynamic of injected drugs [59–61].

For example, in cows, the presence or not of mastitis could have a significant effect on the residual concentration of flunixin in milk, mastitic cows showing slower elimination of the drug [62]. In our study, the C_max_ of penicillin and oxytetracycline in camel milk occurred 24 h after injection. Contrary to penicillin, the residual concentration of streptomycin declined rapidly and reached the MRL (0.2 mg kg^-1^) on average from 30 min after injection. In another example regarding injection of oxytetracycline in dairy goats [63], the mean residence time (MRT) expressing the amount of time spent in the body varied considerably according to the injection route, shorter with IV (3.5h) than with IM (15.3 h) and with IM long action formula (25.0 h).

**Fig 3:**
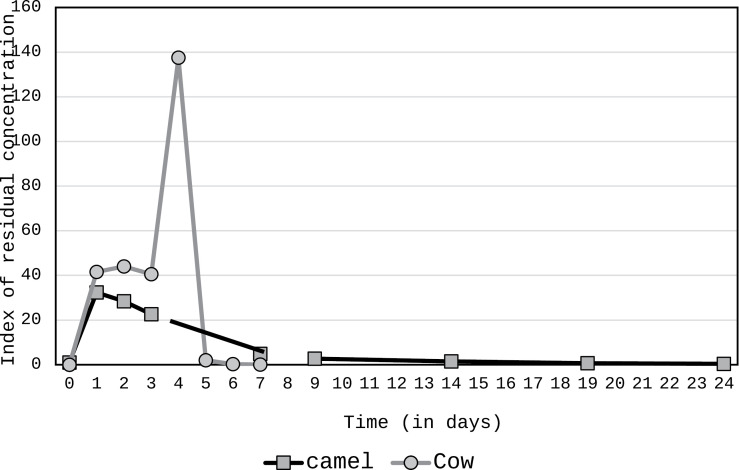
Comparison between relative kinetic (index 1 at t0) of oxytetracycline in camel (our results) and cow [ 51].

Such variability in the experimental conditions leads to questionable comparisons with the literature a fortiori regarding other species. However, regarding penicillin-G intraperitoneal injection in dairy cow, the residue in milk was detected after 31 h (3 milking) at minimum and up to 52 h (5 milking) at maximum, considering that no milk antibiotic residue was observed after 3 days only [64] that is quite shorter than in our case. Indeed, after 24 days, all samples contained oxytetracycline residues between 6 and 33 µg kg^-1^, the mean concentration of residue reached the MRL, i.e., 0.004 mg kg^-1^ for penicillin after 56 days only, correspondingly. After injection of oxytetracycline in healthy dairy cows, the residues in milk reached the MRL after 7 days and was not detectable on the 8th day [50]. Those results supported the withdrawal period in cow milk proposed by the manufacturer, contrary to our results showing that MRL was reached after 2 weeks only in camel milk. Moreover, the comparison of the relative kinetics (at t0, the values were considered as index=1) showed a lower maximum concentration, probably in relationship with the lower antibiotic excretion from blood to milk, and a longer residence time in camel milk (Fig 3). Similar to cow’s buffalo C_max_ for dihydrostreptomycin and penicillin G occur during first milking (~10h) and their levels drop below the maximum residue limit at milkings 12 (~6 days) and 2 (~1 day) respectively [65].

Indeed, another important parameter to determine the risk of milk residue is the rate of antibiotics passing from plasma to milk [66]. Antibiotic excretion into milk from the plasma after injection depends on various factors including the type of compound, physiological status of the animal, milk composition and obviously dairy species. To investigate the transfer from the blood compartment to the udder compartment, a model was developed by Tardiveau et al., (2022) to predict the distribution of oxytetracycline in dairy species, but unfortunately not including camel [67]. As plasma (or serum) concentrations were not determined in our study, it is not possible to have comparison with literature. But the rare comparative references including camel [11] showed a lower ratio milk/plasma of the AUC (Area under concentration *vs* time curve for milk and plasma) after injection of cephalosporin in camel (on average 10.81 and 12.82 for IV and IM injection respectively) compared to goat (18.3 and 14.3 respectively) and over all to cow (47.7 and 51.1 respectively). Thus probably, the risk of antibiotic residues in camel milk could appear confused, as it involves both a lower transfer from blood to milk and a slower elimination rate.

### One health approach

The slow elimination of some antibiotics and their prolonged presence in camel may elevate the risk of antimicrobial resistance development within the camel population and consumers of camel products [68]. Antimicrobial resistance represents an ecological problem impacting the well-being of humans, animals, and their surrounding ecosystem. Huge risks pose antimicrobial resistance genes subtypes with vertical and horizontal transfer possibilities thought the camel products [69]. This underscores the necessity of adopting a One Health approach to comprehensively address the implications of antibiotic usage in camel husbandry. The One Health approach highlights the interdependence of human, animal, and environmental health, emphasizing collaborative efforts across disciplines to effectively manage and prevent health risks. Regulation of prophylactic and treatment methods against camel diseases based on pharmacokinetic and metabolism studies of veterinary drugs in camels will give the possibility to establish effective and safe treatment methods, to prevent zoonotic diseases, infections transmitted naturally from animals to humans and will provide healthy and safe camel products worldwide [12,70,71].

## Conclusions

This pilot study demonstrated that while the concentration of streptomycin in camel milk decreases rapidly and efficiently after injection, the elimination of penicillin and tetracycline is notably slower, even though their transfer to milk is lower compared to other dairy species. These preliminary findings suggest the need to adapt the withdrawal periods specifically for dairy camels rather than relying on data from bovine or other ruminants. Given the limited scope of this study, further research with larger sample sizes and varied conditions is recommended to validate these findings. Additionally, developing specific guidelines for antibiotic use and withdrawal periods in camel milk production is essential to ensure food safety and maintain consumer trust. Especially because the decrease in streptomycin concentration after injection appearing to be rapid and efficient, whereas the elimination of penicillin and tetracycline was slow, the practical recommendations regarding the withdrawal periods should be different between the 3 tested antibiotics.

## Supporting information

S1 File SUPLdata_Antibio in camel milk. (XLSX)
